# Early life social isolation stress and pain vulnerability: unraveling neuroimmune mechanisms and central sensitization

**DOI:** 10.3389/fphar.2026.1844087

**Published:** 2026-06-01

**Authors:** Carmela Belardo, Maria Consiglia Trotta, Federica Ricciardi, Michela Perrone, Andrea Maria Morace, Rebecca Limongelli, Roozbe Bonsale, Erika D’Agostino, Emanuele Di Martino, Antimo Fusco, Silvia Natoli, Francesca Guida, Sabatino Maione, Livio Luongo

**Affiliations:** 1 Department of Life Science, Health and Health Professions, Link Campus University, Rome, Italy; 2 Department of Experimental Medicine, University of Campania “Luigi Vanvitelli”, Naples, Italy; 3 Department of Clinicial-Surgical, Diagnostic and Pediatric Sciences, University of Pavia, Pavia, Italy; 4 Pain Unit, Fondazione IRCSS Policlinico San Matteo, Pavia, Italy

**Keywords:** behaviour, early-life stress, neroimmunity, nociplastic pain, social isolation

## Abstract

Chronic pain is increasingly recognized as a multidimensional condition in which neuroimmune interactions shape disease vulnerability and persistence. Among the emerging pain phenotypes, nociplastic pain (defined as an alteration of nociceptive processing in the absence of clear tissue damage or somatosensory lesion) remains mechanistically elusive and therapeutically challenging. Emerging evidence suggests that early life stress, particularly social isolation, may be a potent psychosocial stressor associated with an increased risk of nociplastic pain. However, this relationship remains incompletely understood and is largely inferred from indirect, model-dependent, or related lines of research. Preclinical and clinical studies indicate that prolonged social isolation can induce sustained activation of the hypothalamic–pituitary–adrenal (HPA) axis, systemic low-grade inflammation, and immune changes. These changes have been shown to promote persistent microglial activation, astrocytic reactivity, and dysregulated neuron–glia communication within pain-processing regions, including the spinal dorsal horn and supraspinal affective circuits. In parallel, peripheral neuroimmune alterations, particularly involving satellite glial cells and Schwann cells may contribute to increased sensory neuron excitability and processes consistent with central sensitization. Notably, much of this evidence derives from studies on stress-related conditions, neuroinflammation, and disorders such as fibromyalgia, rather than being specific to nociplastic pain *per se*. Early life social isolation stress has also been associated with changes in limbic and prefrontal networks implicated in the affective components of pain, suggesting a potential interaction between emotional dysregulation and pain amplification, although causal pathways remain to be clarified. Sex-dependent differences in neuroimmune signalling further add complexity to this framework, suggesting that biological sex may influence vulnerability to isolation-induced pain states and response to glial-targeted interventions. This narrative review proposes a putative neuroimmune model linking early life social isolation stress to nociplastic pain vulnerability and persistence. By integrating evidence from stress biology, glial dysfunction, and central sensitization, we aim to outline a conceptual model that may help guide future research and inform therapeutic strategies targeting central and peripheral neuroinflammatory processes.

## 1 Introduction

**FIGURE 1:**
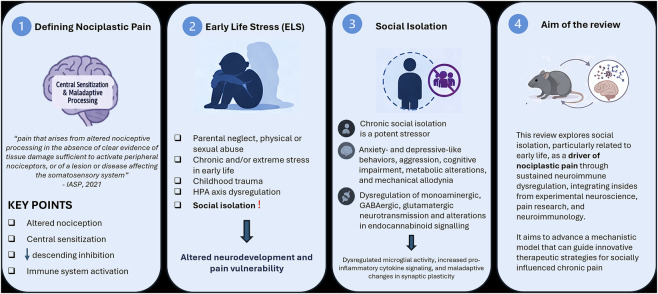
Role of social isolation as a driving factor of nociplastic pain. (1) Nociplastic pain is characterized by altered nociceptive processing in the absence of clear evidence of tissue damage, with central sensitization, reduced descending inhibition, and immune system activation. (2) Early Life Stress (ELS), including neglect, abuse, and childhood trauma, contributes to altered neurodevelopment and increases vulnerability to pain. (3) Chronic social isolation acts as a potent stressor, associated with anxiety- and depressive-like behaviors, cognitive impairment, metabolic alterations, and dysfunction of neurotransmitter systems (monoaminergic, GABAergic, glutamatergic, and endocannabinoid). (4) Together, these factors sustain persistent neuroimmune dysregulation, promoting the development and maintenance of nociplastic pain.

Nociplastic pain represents an emerging concept in pain research, characterized by “*altered nociception without clear evidence of tissue damage or somatosensory system lesion*” (Raja et al., 2020). Nociception is defined as the neural process of encoding noxious stimuli, leading to protective autonomic and behavioural responses (Kosek et al., 2016; Raja et al., 2020). This paradigm challenges traditional distinctions between nociceptive and neuropathic pain, highlighting the role of central sensitization and maladaptive neural processing in chronic pain conditions. Moreover, nociplastic pain frequently coexists with mood disorders, sleep disturbances, and cognitive dysfunction, supporting the presence of shared stress-related pathophysiological mechanisms (Fitzcharles et al., 2021; Kosek et al., 2021). This narrative review addresses several underexplored and debated aspects of nociplastic pain, highlighting social isolation as a potential risk factor that may exacerbate its manifestation (Figure 1).

### 1.1 Defining nociplastic pain: a novel paradigm in pain mechanisms

Chronic pain is among the most prevalent and disabling health conditions worldwide, representing a major socioeconomic burden and significantly impairing quality of life (Kosek et al., 2016; Rice et al., 2016; Raja et al., 2020). This should not be considered solely a peripheral phenomenon, but rather a multifaceted systemic disorder driven by maladaptive interactions between the nervous, endocrine, and immune systems.

Traditionally, pain has been conceptualized as comprising both emotional and sensory dimensions, associated with potential or actual tissue damage (Kosek et al., 2016; Raja et al., 2020). The sensory component has been classified into nociceptive and neuropathic pain. The first type is defined as pain arising from actual or threatened damage to non-neural tissue, caused by the activation of peripheral nociceptors in response to mechanical, thermal, or chemical stimuli. It typically reflects a protective physiological process and is commonly associated with inflammation or tissue injury (Raja et al., 2020). Conversely, neuropathic pain arises from lesions or diseases of the somatosensory nervous system and involves intertwined sensory, emotional, and cognitive components (Majumdar et al., 2026a). It is characterized by altered nociceptive signaling from peripheral to central structures within a distributed pain network, in which coordinated neuroimmune and neuroplastic changes (such as glial activation, pro-inflammatory signaling, and alterations in synaptic and dopaminergic pathways) link peripheral injury to central adaptations (Majumdar et al., 2026a). Notably, emerging evidence suggests that aging significantly influences the development and maintenance of neuropathic pain, potentially through age-related changes in neuroimmune interactions, reduced regenerative capacity, and altered pain processing mechanisms (Majumdar et al., 2026b).

**FIGURE 2:**
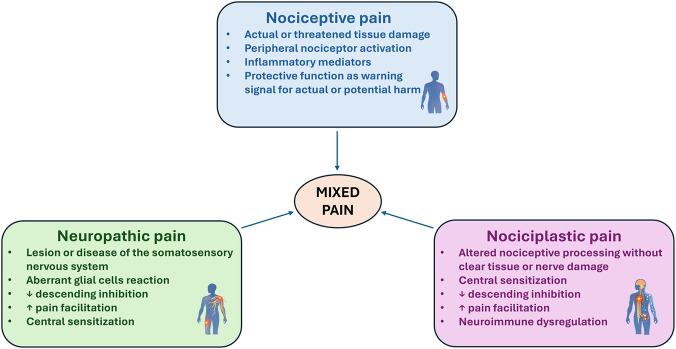
Classification of pain mechanisms: nociceptive, neuropathic, and nociplastic pain. Nociceptive pain arises from actual or threatened tissue injury with a normally functioning nervous system. Neuropathic pain results from damage or disease affecting the somatosensory nervous system and is associated with maladaptive neural changes. Nociplastic pain is characterized by altered nociceptive processing without clear evidence of tissue damage or somatosensory system lesion, leading to amplified pain signaling. These categories may overlap, reflecting the complex and multifactorial nature of pain conditions.

However, the International Association for the Study of Pain (IASP) has proposed the addition of a third category, nociplastic pain, to better define conditions in which altered nociceptive processing occurs without clear nociceptive or neuropathic mechanisms (Table 1). The IASP definition of nociplastic pain refers to *“pain that arises from altered nociceptive processing in the absence of clear evidence of tissue damage sufficient to activate peripheral nociceptors, or of a lesion or disease affecting the somatosensory system”* (Kosek et al., 2021). This concept marks an important shift in the understanding of chronic pain, emphasizing the role of central dysfunction and neuroimmune alterations as key contributors to its persistence (Figure 2).

**TABLE 1:** Different types of pain.

Pain type	Mechanisms	Examples	Treatment
Nociceptive	Tissue damage	Fractures, Trauma, Appendicitis, Muscle injuries	First-line: Paracetamol, NSAIDs Second-line: Opioids (for moderate–severe pain) Adjunctive/local: Topical analgesics, corticosteroids Non-pharmacological: Rest, physiotherapy
Neuropathic	Nerve damage, maladaptive somatosensory processing	Diabetic neuropathy, Trigeminal neuralgia, Multiple sclerosis	First-line: Tricyclic antidepressants, SNRIs, gabapentinoids Second-line: Topical agents (e.g., lidocaine, capsaicin) Other options: Neuromodulation (e.g., spinal cord stimulation)
Nociplastic	No sensory damage, altered pain processing in the central nervous system	Fibromyalgia, Chronic tension-type headache, Irritable bowel syndrome, chronic back pain	Pharmacological: Antidepressants (e.g., TCAs, SNRIs), selected analgesics Non-pharmacological (first-line): Exercise, physiotherapy, cognitive behavioral therapy (CBT), mindfulness Multimodal approach recommended
Mixed pain	Complex interplay between nociceptive, neuropathic and nociplastic pain types	Sciatica, rheumatoid arthritis, cancer pain, endometriosis	Pharmacological: NSAIDs, opioids (when appropriate), adjuvant agents depending on dominant mechanism Other options: Cannabinoids (selected cases) Multimodal approach: Combination of pharmacological and non-pharmacological strategies

Importantly, nociplastic pain requires positive clinical features, including widespread pain distribution, pain hypersensitivity (e.g., hyperalgesia and allodynia), and the presence of symptoms such as fatigue, sleep disturbances, and cognitive dysfunction (Barnes et al., 2021; Fitzcharles et al., 2021; Kosek et al., 2021). These criteria have been further proposed by the IASP to improve diagnostic consistency and to distinguish nociplastic pain from mixed pain states, where chronic primary pain may coexist. Indeed, chronic primary pain represents a broader clinical category characterized by pain persisting for more than 3 months, associated with significant emotional distress or functional disability (Barnes et al., 2021).

A key unresolved issue is whether nociplastic pain can persist independently of ongoing tissue damage due to intrinsic dysfunction in central pain processing. However, this notion is challenged by evidence indicating that sustained central sensitization often depends on continuous nociceptive input from peripheral sources or nerve injury (Woolf, 2011; Treede et al., 2019). Although emerging neuroimaging and quantitative sensory testing (QST) studies suggest that altered central pain modulation can serve as measurable correlates of nociplastic pain, no single biomarker is currently sufficient for diagnosis (Barnes et al., 2021; Fitzcharles et al., 2021).

A critical question arises when pain becomes persistent in the absence of a detectable lesion, particularly when an emotionally salient event represents the precipitating factor in the transition from acute to chronic pain. In such contexts, including psychogenic or affective pain states, it is plausible that peripheral input, even if subtle or not readily measurable, contributes to the initiation and maintenance of the pain experience (Vlaeyen and Linton, 2000; Woolf, 2011). This perspective supports the idea that nociplastic mechanisms provide a neurobiological substrate for symptoms previously attributed primarily to psychological factors (Raja et al., 2020; Kosek et al., 2021).

Among psychosocial stressors, social isolation has emerged as a critical and underrecognized determinant of chronic pain vulnerability (Eisenberger, 2012). Prolonged social deprivation represents a potent stressor capable of inducing profound neurobiological changes. Experimental models of post-weaning social isolation lead to marked neuropsychiatric alterations involving the sensory system, with an amplified perception of harmless stimuli causing increased pain sensitivity (Belardo et al., 2022). These models consistently reproduce behavioral phenotypes relevant to stress-related disorders, including anxiety- and depressive-like behaviors, aggression, cognitive impairment, metabolic alterations, and mechanical allodynia (Fone and Porkess, 2008; Karelina et al., 2009). These alterations are accompanied by dysregulation of monoaminergic, GABAergic, and glutamatergic neurotransmission, as well as profound alterations in endocannabinoid release within brain regions sensitive to stress, such as the medial prefrontal cortex, supporting a mechanistic link between chronic stress exposure, nociplastic pain, and affective dysfunction (Marco, 2011; Jehle et al., 2021).

Therefore, this review explores early life stress (ELS), particularly social isolation, as a driver of nociplastic pain through sustained neuroimmune dysregulation, integrating insights from experimental neuroscience, pain research, and neuroimmunology. It aims to advance a mechanistic model that can guide innovative therapeutic strategies targeting chronic pain influenced by social factors.

### 1.2 ELS: a regulator of emotional behavior and pain vulnerability

During early stages of life, the developing brain is extremely responsive to environmental factors, including stress and childhood trauma (Peña, 2025). Deep alteration in stress-responsive systems, and neural maturation, can lead to long-term changes in emotional behavior and sensory processing (Radley et al., 2015; Aboushaar and Serrano, 2024), predisposing individuals to increased pain vulnerability and neuropsychiatric disorders (Aboushaar and Serrano, 2024). Indeed, adversities in early life such as parental neglect, physical or sexual abuse, and social stress have been associated with a broad spectrum of pathological outcomes, ranging from cognitive decline to increased risk for cardiometabolic syndrome, psychiatric disorders, and chronic pain conditions (Syed and Nemeroff, 2017; Berring et al., 2024). Consistently, individuals suffering from chronic conditions, including chronic pain, post-traumatic stress disorders, major depression and fibromyalgia (defined as a chronic primary pain syndrome of non-inflammatory origin), frequently report a history of trauma during childhood or early adolescence (Nicol et al., 2016; Kosek et al., 2021; Karimov-Zwienenberg et al., 2024).

Importantly, social isolation represents a well-established environmental and experimental ELS subtype, particularly when occurring during critical developmental windows such as the postnatal or juvenile period (Akimova et al., 2009; Lukkes et al., 2009; Grippo et al., 2014). In this regard, the COVID-19 pandemic provided an unexpected naturalistic context of prolonged social restriction and isolation, particularly affecting children and adolescents during sensitive developmental periods. Emerging evidence suggests that pandemic-related social deprivation was associated with increased risk of anxiety, depressive symptoms, sleep disturbances, and stress-related psychopathology in younger populations, thereby reinforcing the role of social isolation as a critical early-life stressor with potential long-term neurodevelopmental consequences (Loades et al., 2020; Racine et al., 2021). These converging experimental and clinical observations support the translational relevance of social isolation as a specific subtype of ELS contributing to later vulnerability to neuropsychiatric and pain-related disorders.

### 1.3 Methods

This narrative review was conducted using a comprehensive, non-systematic literature search aimed at identifying relevant studies on the relationship between social isolation, neuroimmune mechanisms, and nociplastic pain. Databases including PubMed, Scopus, and Web of Science were searched for articles published from 2003 to 2025. The search strategy combined keywords such as “nociplastic pain,” “early life stress,” “social isolation”, “neuroinflammation,” central sensitization,” “microglia,” “astrocytes,” “glial cells,” “Schwann cells,” and “neuroimmune interaction.”

Both preclinical and clinical studies were considered to provide an integrated perspective bridging mechanistic and translational evidence. Studies were selected based on their relevance to the neurobiological and neuroimmune processes linking social isolation to altered nociception. Given the narrative nature of this review, no formal inclusion/exclusion criteria or systematic quality assessment was applied.

## 2 Neuroimmune mechanisms linking ELS to nociplastic pain

There is mounting evidence that immunity pathways are crucial in regulating nociceptive processing in nociplastic pain disorders (Lai et al., 2026). Through intricate neuroimmune interactions, peripheral and central immune cells in the nervous system contribute to the establishment and maintenance of pain. Immune cells, including neutrophils and macrophages in peripheral tissues and sensory ganglia, as well as microglia and astrocytes in the Central Nervous System (CNS), emit pro-inflammatory mediators such as growth factors, chemokines, and cytokines that can directly affect neuronal excitability. Inflammatory mediators and other signaling molecules that can regulate neuronal activity and maintain central sensitization are indeed produced by surveillant glial cells (Ji et al., 2018) interplay between peripheral and central immune processes establishes a feed-forward loop of immunological signals and neuronal hyperexcitability, thereby enhancing the persistence and amplification of pain in nociplastic circumstances.

In this context, ELS has emerged as a critical determinant of long-term immune function and neuroimmune communication. Exposure to psychosocial stress during sensitive developmental periods activates immune signaling pathways and favors the release of circulating pro-inflammatory cytokines, including IL-1β, IL-6, and TNF-α. Although acute stress responses can transiently enhance host defense mechanisms, repeated or chronic stress exposure during early life appears to program immune cells toward a persistent pro-inflammatory phenotype characterized by exaggerated inflammatory responses to later immune or psychosocial challenges (Baumeister et al., 2016; Chiang et al., 2022).

### 2.1 Central mechanisms of neuroimmune dysregulation in ELS

The immune system is highly sensitive to psychological and environmental stressors and usually participates in a dynamic crosstalk with the CNS. Accumulating evidence indicates that exposure to traumatic events, particularly during early life stages, is associated with long-lasting changes in immune function associated with higher levels of pro-inflammatory cytokines such as Interleukin 6 (IL-6) and C-Reactive Protein (PCR) in children with depression and psychosis (Khandaker et al., 2014). Such immune alterations have been linked to an elevated susceptibility to neuropsychiatric disorders later in life (Benjet et al., 2010). Within the CNS, pro-inflammatory signals can influence the activity of resident immune cells, particularly glial cells.

**FIGURE 3:**
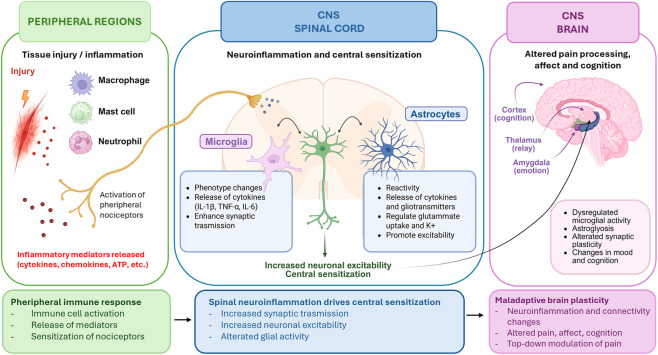
Schematic representation of the neuroimmune mechanisms underlying the transition from peripheral injury to central sensitization and chronic pain. Peripheral tissue injury and inflammation activate immune cells (macrophages, mast cells, and neutrophils), leading to the release of inflammatory mediators that sensitize peripheral nociceptors. Persistent nociceptive input promotes spinal neuroinflammation, characterized by microglial and astrocytic activation, release of pro-inflammatory cytokines, and increased neuronal excitability, ultimately driving central sensitization. At the brain level, maladaptive changes in synaptic plasticity and connectivity within regions involved in sensory, emotional, and cognitive pain processing contribute to the maintenance of chronic pain and its affective and cognitive dimensions.

Microglia, known as the resident immune cells of the CNS, are highly dynamic phagocytic cells that colonize the brain during early development and continuously survey the neural environment, contributing to the removal of cellular debris and maintenance of homeostasis (Colonna and Butovsky, 2017) (Figure 3). Beyond their classical role in immune surveillance, microglia are recognized as key players of neuroinflammatory modulation and exert an essential role in shaping synaptic plasticity as well as the maturation and remodeling of neural circuits (Paolicelli et al., 2011; Calcia et al., 2016). Several studies described the role of microglia in modulating the development of pain-related phenotypes following early life stress. Exposure to traumatic experiences during critical developmental age can alter microglial maturation in male mice, leading to a long-lasting primed state, characterized by an augmented response to subsequent stimuli (Diz-Chaves et al., 2013). In these animals, dysregulated microglial activity may contribute to persistent neuroinflammatory cross-talk within brain regions involved in pain perception and modulation, such as the hippocampus, prefrontal cortex, and amygdala (Reemst et al., 2022). These alterations can facilitate maladaptive plasticity within pain-involved circuits. ELS-induced microglial changes are considered an important mechanistic link between environmental adversity and augmented susceptibility to chronic and nociplastic pain conditions later in life. In the end, ELS can alter the normal developmental trajectory of microglia, affecting processes such as proliferation, survival, and phagocytic function in male mice (Reemst et al., 2022). Since microglia actively contribute to the physiological state of neural circuits during important developmental windows, adversity in their maturation may also influence a variety of neurodevelopmental processes. Consequently, exposure to a critical stressor during early life may lead to persistent changes in microglial phenotype that ultimately impact brain development and later behavioral outcomes (Catale et al., 2020).

Astrocytes, the most numerous CNS glial cell type, play a central role in regulating neuronal homeostasis and coordinating the complex communication among neural, metabolic, and glial signals (Gradisnik and Velnar, 2023) (Figure 3). In addition to these functions, astrocytes actively regulate synaptic transmission, extracellular ion balance, and metabolic communication between neurons and glial cells (Miguel-Hidalgo, 2022). A key function in astrocytic life is the control of glutamatergic neurotransmission: astrocytic processes surrounding synapses express high levels of glutamate transporters, such as EAAT1 and EAAT2 (GLT-1), which clear glutamate from the extracellular space and convert it into glutamine via glutamine synthetase, thereby sustaining the glutamate–glutamine cycle that supports neuronal signaling (Depaauw-Holt et al., 2025). Several pieces of evidence indicate that ELS can alter astrocyte physiology and their interactions with the neuroimmune environment. Astrocytes express receptors on the cellular membrane for cytokines and chemokines, such as IL-1R1, TNFR, IL-6R, and CCR1; for this reason, human astrocytes are highly responsive to inflammatory mediators released by other actors in the CNS parenchyma, like microglia or infiltrating peripheral immune cells (Dorf et al., 2000; Choi et al., 2014). Through these pathways, astrocytes can alter the expression of immune mediators and chemokines, potentially amplifying neuroimmune responses and influencing neuronal cells’ function. Stress-related immune activation can consequently affect several astrocytic functions essential for neuronal activity, including neurotransmitter metabolism, glutamate transport, gap junction communication, and regulation of the extracellular environment. Exposure to stress during critical developmental stages appears to induce, similar to microglia involvement, persistent alterations in astrocyte structure and function (Miguel-Hidalgo, 2022). Preclinical models of ELS, such as maternal separation in rodents, have shown reductions in astrocyte density and decreased expression of astrocytic markers such as GFAP and S100β in brain regions sensitive to stress, including the amygdala, prefrontal cortex and hippocampus (Abbink et al., 2019). These changes are often accompanied by reduced morphological complexity of astrocytic processes and impaired uptake of glutamate and GABA, suggesting a diminished capacity of astrocytes to regulate synaptic transmission and maintain neurotransmitter homeostasis. Early adversity, at the molecular level, has also been associated with dysregulation of astrocytic glutamate transporters, particularly GLT-1, resulting in reduced glutamate clearance and increased neuronal excitability in several brain regions implicated in stress responses, including the cortex, hippocampus, hypothalamus, and habenula (Miguel-Hidalgo, 2022). In addition, astrocytes are strategically positioned to detect circulating glucocorticoids due to their close association with cerebral vasculature, allowing stress hormones to directly influence astrocytic signaling pathways and gene expression. Together, these observations suggest that early-life stress can modify astrocyte-mediated regulation of synaptic and neuroimmune processes, potentially contributing to long-term alterations in neuronal circuit function.

### 2.2 Peripheral mechanisms of neuroimmune dysregulation in ELS

Growing evidence indicates that immune reprogramming primarily involves cells of the myeloid lineage. Transcriptomic studies showed that exposure to childhood trauma is associated with differential gene expression within circulating immune cells, particularly monocytes and dendritic cells, suggesting that early stress can reshape innate immune regulatory networks modulating inflammatory gene expression (Cole et al., 2012; Bower, 2019) (Figure 3). Consistent with these findings, genome-wide transcriptional profiling analyses have demonstrated that adolescents exposed to childhood adversity display distinct transcriptional signatures within circulating leukocytes, with differential gene expression patterns originating predominantly from myeloid-lineage immune cells, including dendritic cell subsets and non-classical monocytes (Kuhlman et al., 2023; Kuhlman et al., 2025). These alterations are paralleled by modifications in transcription factor activity within immune regulatory pathways, including interferon regulatory factors (IRFs) and CREB signaling, which modulate inflammatory gene expression, leukocyte recruitment, and immune activation. Together, these findings suggest that early adversity can induce durable immune genomic programming, potentially contributing to long-term immune dysregulation and altered inflammatory reactivity across the lifespan.

Importantly, peripheral immune alterations induced by early stress may also influence neuroimmune communication and pain vulnerability. Peripheral immune mediators can interact with primary sensory neurons and glial cells, modulating nociceptive processing through cytokine release, chemokine signaling, and immune–neural interactions within sensory ganglia. In this context, accumulating evidence indicates that immune dysregulation plays a role in nociplastic pain conditions, including fibromyalgia. Passive transfer studies have shown that IgG isolated from individuals with fibromyalgia can elicit mechanical and cold hypersensitivity in mice. Notably, these patient-derived antibodies localize to dorsal root ganglia (DRG) structures, such as satellite glial cells and sensory fibers, thereby modulating sensory neuron function (Goebel et al., 2021). More recently, studies have further shown that neutrophils derived from patients with fibromyalgia can infiltrate sensory ganglia and induce mechanical hypersensitivity when transferred into naïve mice, supporting a direct pronociceptive role of peripheral immune cells in modulating sensory neuron excitability and chronic widespread pain (Caxaria et al., 2023).

Collectively, these observations support the concept that early-life stress may promote long-lasting immune remodeling that extends beyond classical inflammatory responses, influencing peripheral neuroimmune signaling and potentially increasing susceptibility to chronic pain disorders later in life.

### 2.3 Neuroimmune and glial mechanisms underlying nociplastic pain phenotypes

While microglial, astrocytic, and peripheral immune alterations induced by ELS are increasingly well characterized, their mechanistic relevance becomes particularly evident when considered across major human nociplastic pain conditions. Specifically, converging evidence in fibromyalgia supports central sensitization driven by glial dysregulation and altered descending pain modulation, alongside peripheral immune contributions that may further enhance dorsal root ganglia excitability (Carneiro et al., 2025). In irritable bowel syndrome, neuroimmune interactions within the gut–brain axis, including mast cell–neuronal crosstalk and enteric glial activation, have been implicated in visceral hypersensitivity and altered central processing of visceral afferents (Ganesh et al., 2019). In chronic primary low back pain, maladaptive neuroimmune signaling within spinal and supraspinal networks has been associated with sustained pain amplification and altered central processing, even in the absence of clear structural pathology, suggesting a key role for glial and immune dysregulation in maintaining chronicity (Pinheiro et al., 2026). Finally, in tension-type headache, altered central pain processing together with evidence of neuroimmune modulation of trigeminal and pericranial afferents supports a role for low-grade neuroinflammation in headache chronification (Burshtein et al., 2015). Collectively, these observations provide a unifying framework in which ELS-induced neuroimmune programming may lower the threshold for the emergence and persistence of nociplastic pain across multiple clinical phenotypes.

## 3 Social isolation as a chronic stressor for nociplastic pain vulnerability?

Stress adaptation represents an essential biological function for the survival of organisms. The response to stressors involves a complex interaction among the nervous, endocrine, and immune systems, and the inability to effectively adapt to prolonged stress conditions represents an important risk factor for numerous diseases (Yusuf et al., 2025). Several studies demonstrate that stressful experiences occurring early in life, such as social isolation, can significantly influence brain development and behavior (Weiss et al., 2004).

Social isolation is considered relevant for several psychiatric conditions, including post-traumatic stress disorder (PTSD) (Pearce et al., 2023). Numerous studies support the reliability of this paradigm, as it induces persistent behavioral and neurobiological alterations like PTSD, including increased anxiety, cognitive deficits, social withdrawal, and heightened stress reactivity. These alterations are associated with long-lasting changes in neural circuits involved in stress regulation, including dysregulation of the hypothalamic–pituitary–adrenal (HPA) axis, alterations in monoaminergic transmission, and changes in functional coupling between the amygdala and prefrontal cortex (Ricciardi et al., 2025).

**FIGURE 4:**
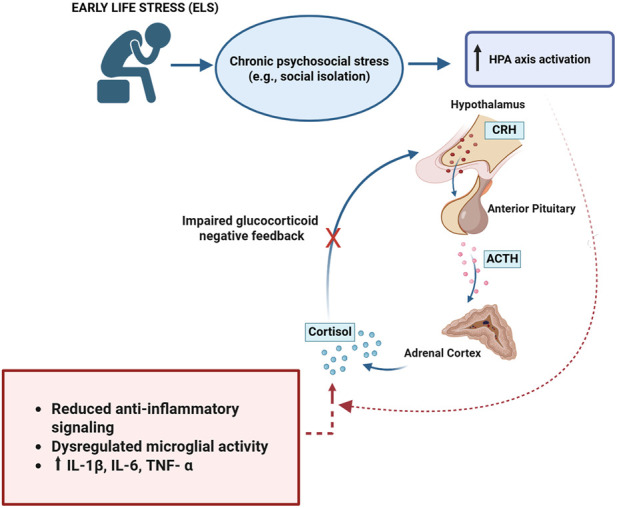
Social isolation–induced activation of the hypothalamic–pituitary–adrenal (HPA) axis and its immunological consequences. Chronic social isolation is a persistent stressor, that leads to sustained activation of the HPA axis. This involves hypothalamic release of corticotropin-releasing hormone (CRH), stimulation of the anterior pituitary to secrete adrenocorticotropic hormone (ACTH), and subsequent cortisol production from the adrenal cortex. Prolonged activation disrupts normal negative feedback regulation of cortisol, resulting in impaired anti-inflammatory control and increased production of pro-inflammatory cytokines (e.g., IL-1β, IL-6, TNF-α) and dysregulated microglial activity. This neuroendocrine–immune dysregulation may contribute to the development and maintenance of chronic pain states. IL-1β: Interleukin 1 beta; IL-6: Interleukin 6; TNF-α: Tumor Necrosis Factor-alpha.

The HPA axis represents one of the main neuroendocrine systems involved in the stress response (Figure 4). Under physiological conditions, a stressful stimulus activates neurons in the paraventricular nucleus (PVN) of the hypothalamus, inducing the release of vasopressin and corticotropin-releasing hormone (CRH). These mediators induce secretion of adrenocorticotropic hormone (ACTH) from the anterior pituitary, thereby promoting the production of glucocorticoids by the adrenal cortex. Glucocorticoids subsequently exert negative feedback on the HPA axis by modulating the neuronal activity in the hippocampus and the PVN, overall reducing the secretion of CRH and ACTH (Herman et al., 2012). Glucocorticoids play a fundamental role in several physiological processes, including energy metabolism, glucose homeostasis, and neuronal survival. Their altered levels have been associated with the development of stress-related pathologies, including depression and metabolic syndrome (Vargas et al., 2016). Once released into circulation, glucocorticoids modulate emotional behavior and stress responses by activating two main types of intracellular receptors: mineralocorticoid receptors (MR) and glucocorticoid receptors (GR), which act as transcription factors that regulate gene expression. In addition to genomic effects, corticosteroids can also exert rapid non-genomic actions at neuronal membranes, contributing to the modulation of vigilance and risk-assessment behaviors (Reis et al., 2012). Corticosterone-associated risk assessment behaviors may contribute to the defensive response to social isolation in rats, possibly via glucocorticoid receptor activation and with differences depending on sex and developmental stage (Reis et al., 2012; Pisu et al., 2016; Boero et al., 2018). In social isolation models, significant alterations in glucocorticoid receptor sensitivity have been described. An upregulation of GR immunoreactivity in the hypothalamus and hippocampus has been observed, associated with stress-induced neuronal loss and reduction in hippocampal volume. Conversely, MR receptors are often downregulated, reducing resilience to subsequent stress events (Vlachos et al., 2020). While HPA axis dysregulation is well established in stress-related disorders, and PTSD is consistently linked to decreased cortisol levels, significant individual differences in this response are still observed. Cortisol levels are shaped by several biological and environmental factors, such as age, circadian regulation, cumulative stress exposure, and individual clinical background; however, sex and the nature of trauma exposure appear to be particularly important determinants (Paris et al., 2010; Flandreau and Toth, 2017; Sterina et al., 2022).

In addition to neuroendocrine effects, chronic stress associated with social isolation significantly impacts on the immune system. Clinical and experimental evidence indicates that chronic stress promotes a systemic inflammatory state characterized by an increase in circulating pro-inflammatory immune cells, particularly monocytes, contributing to the development of anxiety states and behavioral alterations (Biltz et al., 2022). Moreover, the detection of pathogen-associated molecular patterns (PAMPs) and damage-associated molecular patterns (DAMPs) by pattern recognition receptors (PRRs) expressed on innate immune system cells (dendritic cells, macrophages and neutrophils) may favor the activation of the transcription factor NF-κB (Nuclear Factor kappa-light-chain-enhancer of activated B cells) (Al Omran et al., 2022; Matthews et al., 2024; Qaisiya et al., 2025). When activated, NF-κB translocates to the nucleus and promotes transcription of pro-inflammatory genes, such as IL6, by binding to their promoter regions. This transcriptional activity also contributes to macrophage polarization toward a pro-inflammatory M1-like phenotype, characterized by the production of cytokines such as IL-1β, IL-6, and TNF-α (Liu et al., 2017; Omran et al., 2021).

Overall, these findings suggest that chronic social isolation does not act solely as a psychological stressor but represents a powerful neuro-immuno-endocrine modulator capable of inducing persistent activation of the HPA axis and a state of systemic inflammation, contributing to the so-called immune priming and increasing vulnerability to the development of neuropsychiatric disorders and inflammatory diseases.

### 3.1 Early-life adversities: social isolation and stress. Implications for affective circuits and disease vulnerability

Early life environment represents a sensitive developmental window critical for human maturation, with severe repercussions on long-term health (Chen and Baram, 2016; Luby et al., 2020). Both human and rodent studies indicate that brain development extends from prenatal stages into postnatal life (Watson et al., 2006). During this period, early modifications in neural structures and functions shape developmental trajectories and ultimately impact health outcomes in adulthood (Marzola et al., 2023). Notably, the timing of the developmental process determines region-specific vulnerability (Rice and Barone, 2000; McQuillen and Ferriero, 2004; Seidlitz et al., 2020). Brain areas, such as the hippocampus, cortex and cerebellum (which undergo relatively late maturation) seem to be particularly sensitive to early-life perturbations. Although rodents and humans do not share a completely overlapping neurodevelopmental timeline, studies have demonstrated that maturation trajectories in rodents and humans can be aligned through translational time windows, allowing meaningful cross-species comparison (Callaghan et al., 2019; Gegenhuber and Tollkuhn, 2020; Luby et al., 2020). Indeed, although not chronologically aligned, they are comparable developmental sequence. Processes that occur prenatally in humans, including cortical development and neuronal migration, often take place postnatally in rodents. This “temporal translation” is crucial for the use of rodents as valid preclinical models, particularly for investigating conserved neurobiological processes underlying stress responses and brain development, provided that developmental equivalence is considered (Andersen, 2003).

Among the various forms of early-life adversity, stress has emerged as a major determinant of long-term outcomes in humans. Physiologically, stress represents a disruption of homeostasis that engages mainly the HPA axis, as well as the cardiovascular, immune, and metabolic systems (Mariotti, 2015). During periods of intense neurodevelopmental plasticity, these physiological changes may become maladaptive rather than protective (Miczek et al., 2008; Radley et al., 2015).

Indeed, sustained activation of the HPA axis can result in alterations of synaptic plasticity, and structural remodeling within brain regions sensitive to stress, such as the amygdala, hippocampus, and prefrontal cortex (Lucassen et al., 2014). The endocrine activation of the same axis contributes to long-term modulation of limbic and prefrontal pathways, whereas rapid neural circuits mediate the immediate stress response (Ulrich-Lai and Herman, 2009). Moreover, stress-induced activation of the HPA axis also leads to the alteration of stress-related gene expression in the paraventricular nucleus of the hypothalamus changes (Schmidt et al., 2003).

Several lines of evidence indicate that ELS affects subcortical limbic regions and the frontal cortical circuits, mostly when stressful stimuli occur during sensitive time frames of brain maturation in women (Kaiser et al., 2018). Notably, the medial prefrontal cortex (mPFC), is the brain region that takes longer to complete structural and functional maturation, making it particularly vulnerable to early environmental perturbations. Alterations within these circuits in humans and rodents can disrupt cognitive and emotional processing and, reward pathways (Russo and Nestler, 2013). In this context, the mesolimbic circuitry plays a key role. The Ventral Tegmental Area (VTA) projects dopaminergic inputs to the striatum, particularly to the nucleus accumbens (NAc), as well as other forebrain areas. The NAc integrates dopaminergic input from the VTA with glutamatergic projections from these forebrain regions (mPFC, amygdala, and hippocampus), combining executive signals, emotional, and contextual information. Dysregulation of these dopaminergic-glutamatergic systems mediated by ELS in mice seems to be linked also to stress-induced microglia activation, are strongly linked to the development of affective and addictive disorders (Bagot et al., 2015).

Prefrontal cortex-amygdala circuits are critical for the regulation of social behavior. Indeed, bidirectional circuits between infralimbic cortex and basolateral amygdala regulate social interaction; meanwhile prelimbic projections to basolateral amygdala or NAc reduce social attitude. Interestingly, early life experiences in mice have been shown to shape additional pathways, including the PFC-Paraventricular Thalamic pathway, which mediates social behaviors in adulthood (Yamamuro et al., 2020; Li et al., 2025). Indeed, sociability is a dynamic event that shapes and activates peculiar cerebral pathways. The amygdala serves as a crucial hub for preserving social homeostasis. Distinct neuronal populations within the medial, basolateral, and central nuclei of the amygdala play essential roles in encoding social behaviour. A combination of optogenetics and activation of behaviorally salient cells in the central amygdala showed the existence of a hub of social cell that selectively encode social signals, which do not overlap with food reward processing (Madeira and Márquez, 2023; Willmore et al., 2023).

The habenula has recently emerged as an important hub in the regulation of social behavior. In particular, the lateral habenula (LHb) exerts top-down regulation of monoaminergic systems and is responsive to unexpected rewarding or aversive stimuli, as well as to environmental signals that predict potential rewards or punishments (Mondoloni et al., 2022). Consistent with its role in aversive processing, preclinical evidence shows that social isolation increases c-fos expression in the habenula in an exposure duration-dependent manner, suggesting that this structure is recruited during negative emotional states associated with social deprivation (Van Kerkhof et al., 2013). Moreover, the habenula interacts with mesolimbic dopaminergic circuits involved in the social modulation of pain. Recent studies indicate that glutamatergic projections from VTA to the NAc shell and to the lateral habenula form segregated pathways that differentially regulate empathic pain behaviors. Specifically, the VTA–NAc projection promotes resilience to empathic pain, whereas the VTA–LHb pathway contributes to the maintenance of aversive pain-related states (Sha et al., 2024).

Advances in human neuroimaging has revealed that perceived loneliness and social isolation affect wide-range brain networks. Indeed, these conditions are associated with structural and functional alterations within default mode network, defined by cortical structures such as prefrontal cortex, cingulate, precuneus, angular gyrus and hippocampus, and white matter dysregulation (Mak et al., 2017). Several studies shows that social isolated individuals appear to rely more on internally focused thinking and imagined social interactions (Spreng et al., 2020).

Perceived social isolation has been also linked to a pro-inflammatory transcriptional profile characterized by increased NF-κB activity and reduced lymphocyte sensitivity to physiological regulation by the HPA axis (Cacioppo and Hawkley, 2009). Stressors, like deprivation of social bond during early life priming microglia responses to future challenges, may accelerate the effect of senescence on microglia, leading also to premature loss of or alteration in microglia physiological roles (Catale et al., 2022). Moreover, preclinical model of social isolation showed that early social stressors, can alter microglial number, morphology, and motility, particularly in regions sensitive to stress such as the ventral tegmental area and the hippocampus (Shin and Kim, 2023). In addition to microglial priming, stress-induced immune activation also engages astrocytes, which react to inflammatory signals and enhance neuroimmune communication via NF-κB–dependent pathways (Miguel-Hidalgo, 2022).

Inflammation triggered by ELS is associated with the emergence of characteristic behavioral phenotypes, including heightened anxiety, depressive-like behaviors, and increased vulnerability to stress (Shin and Kim, 2023). Converging evidence from clinical and preclinical studies indicates that such early perturbations increase vulnerability to a broad spectrum of disorders, including neuropsychiatric conditions (e.g., depression, anxiety, substance use, post-traumatic stress disorders), neurosensory alterations (e.g., hyperalgesia), and metabolic disturbances (e.g., obesity, insulin resistance) which often persist into adulthood (Butler and Finn, 2009; Heim and Binder, 2012; Mariotti, 2015).

### 3.2 Preclinical models of social isolation during ELS

Several preclinical models, including pharmacological models (Neal et al., 2004; Harré et al., 2008; Wagner et al., 2011), have been developed to mimic and analyze the impact of ELS, such as maternal separation, prenatal stress, limited bedding, social defeat, and social stress (Schmidt et al., 2003; Guzman et al., 2016; Huang et al., 2023). Chen and Baraa highlighted how ELS can reprogram cognitive and emotional brain networks (Chen and Baram, 2016), and how rodent models have been instrumental in identifying both vulnerability and resilience factors (Murthy and Gould, 2018).

Social isolation during the post-weaning period in rodents is one of the most validated models to investigate the long-term consequences of ELS (Andersen, 2003; Semple et al., 2013). This model has been shown to induce anxiety and depressive-like behaviors, altered coping strategies, and changes in neurosensory circuits (Belardo et al., 2022; Ricciardi et al., 2025).

Applications of social isolation protocols in rodents highlight a wide methodological variability, which influences behavioral and neurobiological outcomes observed. Across studies, these protocols are mainly applied in mice and rats, with commonly used strains including CD1 and C57BL/6J mice and Sprague-Dawley or Wistar rats, although strain-dependent differences in stress susceptibility and behavioral responses have been consistently reported (Rivera-Irizarry et al., 2020; Kinley et al., 2021) (Table 2).

**TABLE 2:** Social isolation protocols.

References	Species-strain	Sex	Isolation onset	Duration
Kinley et al. (2021)	Long evans rats	male and female	PND 21	3 weeks
Rivera-Irizarry et al. (2020)	C57BL/6J MICE	male and female	PND28 SI adolescence protocol and PND77 SI adulthood protocol	6 weeks
Challa et al. (2023)	C57BL/6J MICE	male and female	PND 21	8 weeks
Smolensky et al. (2024)	C57BL/6J MICE	male and female	13 Weeks old	4 weeks
Nakayama and Miyata (2025)	C57BL/6N (WILD-TYPE) C57BL/6J TRANGENIC MICE [female just as supplementary experiments]	male	PND21 SI adolescence protocol and PND56 SI adulthood protocol	≥5 weeks with additional 4 weeks resocialization
Al Omran et al. (2022)	C57BL/6J MICE	male	8 Weeks old	4 weeks
Lallai et al. (2024)	Sprague dawley rats	male	PND21	≈3-6weeks
Evans et al. (2012)	Wistar rats	male	PND30	6 weeks
Belardo et al. (2022)	CD1 MICE	male	PND21	30–120 days
Ricciardi et al. (2025)	CD1 MICE	male	PND21	4 weeks

In terms of sex, most studies have historically focused on male animals; however, increasing evidence indicates sex-dependent effects of social isolation, with females often displaying distinct behavioral and neuroimmune profiles or no outcomes, stressing the importance of including both sexes in experimental designs (Challa et al., 2023; Smolensky et al., 2024). Notably, several lines of evidence indicate that differences in species, strain, sex, timing, and duration of isolation can lead to divergent behavioral, neurochemical, and neuroimmune phenotypes, including variability in anxiety- and depressive-like behaviors, stress reactivity, and inflammatory responses (Al Omran et al., 2022; Nakayama and Miyata, 2025). Moreover, it should be noted that the number of animals used in group-housed control groups is often variable, meaning that outcomes may be compared across different behavioral baselines influenced by the social dynamics and bonding of control animals.

A key variable is the developmental timing choosen for the application of isolation protocols. In preclinical models of early-life stress, social isolation is typically initiated during the post-weaning period, precisely postnatal day (PDN) 21–28, corresponding to a critical window of brain maturation and social development (Andersen, 2003; Semple et al., 2013; Torres-Berrío et al., 2026). Moreover, some protocols extend isolation into adolescence or adulthood, leading to partially overlapping but distinct phenotypic outcomes (Lallai et al., 2024). The duration of isolation also varies widely across studies, ranging from short-term paradigms (1-2 weeks) to chronic isolation lasting several weeks (4-8 weeks or longer), with longer exposure generally associated with more robust and persistent alterations in emotional behavior, reward processing, and neuroimmune signaling (Matsumoto et al., 2019; Lallai et al., 2024).

In addition, housing conditions and protocol-specific factors, such as complete individual housing versus limited social interaction, environmental enrichment, and handling procedures, further contribute to variability in outcomes (Matsumoto et al., 2005). Consistently, post-weaning social isolation has been shown to induce alterations in emotional and coping behaviors, as well as in neurosensory processing and pain-related circuits, further supporting its relevance as a model to investigate ELS long-term consequences (Belardo et al., 2022; Ricciardi et al., 2025).

Overall, the current evidence reported effectively highlights methodological heterogeneity and the lack of standardization across studies, with resulting difficulty in directly comparing findings across experimental paradigms. Importantly, these isolation models do not capture a single unified ELS construct, but rather isolate specific dimensions such as social deprivation, developmental timing, or chronicity of stress exposure. As a consequence, differences in species, strain, sex, and protocol design may lead to partially distinct neurobiological adaptations, which should not be over-interpreted as equivalent manifestations of the same underlying pathology. In addition, the specific aims of each study strongly influence the selection of behavioral, neurochemical, or neuroimmune endpoints, further contributing to heterogeneity in the reported findings. This variability makes it challenging to establish standardized guidelines for the application of social isolation paradigms across studies. Nevertheless, despite this heterogeneity, social isolation remains a highly reliable and widely validated preclinical model for investigating the long-term sequelae of ELS.

## 4 Sexual dimorphism in nociplastic pain and its possible role in social isolation

Stressors in early life stages interact with biological and psychosocial pathways in a sex-dependent manner, for example, predisposing females to depression and anxiety, while exacerbating aggressive behavior in male rodents (Oliveira et al., 2024; Takahashi, 2025). This distinction appears to be caused by direct hormone interactions, along with differences in immunological signaling and neural plasticity, which have implications at both the peripheral and central levels (Gregus et al., 2021). A recent study by Laham et al. in mice, (Laham et al., 2022), supports the view that estrous cycle phases should not be considered as a direct causal determinant of behavioral outcomes, but rather biological modulators influencing phenotypic expression. The same authors (Laham et al., 2022) showed that the diestrus phase can be considered an “unmasking phase”’ during which alterations induced by early life adversity become more readily detectable, highlighting that hormonal state may modulate rather than directly drive behavioral outcomes.

A growing body of research shows that sex dimorphism is also present in the transition from acute to chronic pain. In both preclinical and clinical studies, females appear to have a higher prevalence of chronic painful disorders, increased pain sensitivity, and a more difficult recovery from injury than males. This distinction appears to be caused by direct hormone interactions, neuroendocrine mechanisms (Taylor, 2010; White and Kaffman, 2019), along with differences in immunological signaling and neural plasticity, which have implications at both the peripheral and central levels (Gregus et al., 2021).

### 4.1 Microglial differences between males and females

Several neurodevelopmental processes regulated by microglia exhibit marked sexual dimorphism in the rodent brain, suggesting that perinatal hormonal differences and epigenetic mechanisms contribute to stabilizing a sex-specific immune “set-point” (Forger, 2006; Schwarz and McCarthy, 2008).

Consistent with this hypothesis, morphometric analyses have demonstrated that these differences are region- and stage-dependent. In brain regions like the amygdala, hippocampus, and prefrontal cortex (PFC), sex-specific variations in microglial density and morphology have been described during development, whereas in the paraventricular nucleus of the hypothalamus (PVN) these differences appear transient and limited to the neonatal period (Schwarz et al., 2012). This spatial and temporal dimorphism suggests that males and females follow divergent microglial developmental trajectories that may differentially influence responses to injury, inflammation, or stress.

More recent evidence indicates that microglial maturation, transcriptional programming, and functional responsiveness significantly diverge between male and female, generating a sexually dimorphic neuroimmune landscape already during early developmental stages (O’Connor and Nissen, 2023). Consequently, identical perturbations of CNS homeostasis may activate distinct neuroimmune circuits depending on biological sex.

## 5 Conclusion

In conclusion, accumulating evidence indicates that nociplastic pain should not be viewed solely as a disorder of altered nociceptive transmission, but rather as the outcome of complex interactions between chronic stress, neuroimmune dysregulation, and maladaptive plasticity within central and peripheral circuits. Within this framework, early life social isolation stress emerges as a potent risk factor that induces long-lasting alterations in stress-response systems and immune function, ultimately promoting inflammatory priming and increased susceptibility to central sensitization. The presence of sex-dependent differences in neuroimmune pathways further highlights the need for a more personalized understanding of pain mechanisms and treatment strategies. In murine models, the predominant involvement of T cells in females (Sorge et al., 2015) suggests that modulation of adaptive immunity may represent a critical mechanism underlying female vulnerability to persistent pain phenotypes consistent with nociplastic pain. The existence of alternative, sex-specific neuroimmune circuits implies that the same pathological stimulus may be processed through different pathways in males and females. Furthermore, the microglial dimorphism described during development (Atta et al., 2023; O’Connor and Nissen, 2023) may predispose males and females to distinct inflammatory responses and patterns of circuit plasticity in adulthood, potentially contributing to the higher prevalence of nociplastic syndromes in women.

Despite significant advances, important challenges remain, including the limited translation of preclinical findings into clinical contexts and the lack of reliable biomarkers for nociplastic pain. Future research should therefore adopt integrative and multidisciplinary approaches to elucidate causal mechanisms better and to develop innovative therapeutic strategies that account not only for biological processes but also for psychosocial determinants of chronic pain.
